# Hepatic arterial infusion of autologous CD34^+^ cells for hepatitis C virus-related decompensated cirrhosis: A multicenter, open-label, exploratory randomized controlled trial

**DOI:** 10.1016/j.reth.2024.04.018

**Published:** 2024-05-04

**Authors:** Toru Nakamura, Atsutaka Masuda, Makoto Kako, Hirayuki Enomoto, Masaki Kaibori, Yasuyuki Fujita, Kyoko Tanizawa, Tetsuya Ioji, Yoshihiro Fujimori, Kei Fukami, Takuma Hazama, Hideki Iwamoto, Yasukazu Kako, Kaoru Kobayashi, Hironori Koga, Koji Nagafuji, Takayasu Ohtake, Hiroyuki Suzuki, Tomoyuki Takashima, Toshitaka Tsukiyama, Haruki Uojima, Kenichi Yamahara, Koichiro Yamakado, Hidekazu Yamamoto, Kazunori Yoh, Satoshi Yoshihara, Atsuhiko Kawamoto, Shuhei Nishiguchi, Shuzo Kobayashi, Takuji Torimura, Takumi Kawaguchi

**Affiliations:** a Division of Gastroenterology, Department of Medicine, Kurume University School of Medicine, Kurume, Fukuoka, 8300011, Japan; b Liver Cancer Research Division, Research Center for Innovative Cancer Therapy, Kurume University, Kurume, Fukuoka, 8300011, Japan; c Gastroenterology Medicine Center, Shonan Kamakura General Hospital, Kamakura, Kanagawa, 2478533, Japan; d Division of Hepatobiliary and Pancreatic Diseases, Department of Gastroenterology, Hyogo Medical University, Nishinomiya, Hyogo, 6638501, Japan; e Department of Surgery, Kansai Medical University, 2-5-1 Shinmachi, Hirakata, 5731191, Japan; f Translational Research Center for Medical Innovation, Foundation for Biomedical Research and Innovation at Kobe, Kobe, Hyogo, 6500047, Japan; g Department of Transfusion Medicine and Cellular Therapy, Hyogo Medical University, Nishinomiya, Hyogo, 6638501, Japan; h Division of Nephrology, Department of Medicine, Kurume University School of Medicine, Kurume, Fukuoka, 8300011, Japan; i Department of Radiology, Hyogo Medical University, Nishinomiya, Hyogo, 6638501, Japan; j Department of Radiology, Kawanishi City Medical Center, Kawanishi, 6660017, Japan; k Division of Hematology and Oncology, Department of Medicine, Kurume University School of Medicine, Kurume, Fukuoka, 8300011, Japan; l Department of Regenerative Medicine, The Center for Cell Therapy & Regenerative Medicine, Shonan Kamakura General Hospital, Kamakura, Kanagawa, 2478533, Japan; m Department of Radiology and Interventional Radiology, Shonan Kamakura General Hospital, Kamakura, Kanagawa, 2478533, Japan; n Department of Genome Medical Sciences Project, Research Institute, National Center for Global Health and Medicine, Ichikawa, Chiba, 2728516, Japan; o Laboratory of Molecular and Cellular Therapy, Institute for Advanced Medical Sciences, Hyogo Medical University, Nishinomiya, Hyogo, 6638501, Japan; p Yoh Digestive Clinic, Wakayama, 6408269, Japan; q Department of Gastroenterology, Kano General Hospital, Osaka, Japan, 5310041, Japan; r Department of Kidney Disease and Transplant Center, Shonan Kamakura General Hospital, Kamakura, Kanagawa, 2478533, Japan; s Department of Gastroenterology, Omuta City Hospital, Omuta, 8368567, Japan

**Keywords:** CD34 antigens, Cell therapy, Clinical trial, Cirrhosis

## Abstract

**Introduction:**

In this multicenter clinical study, we aimed to investigate the efficacy and safety of the transhepatic arterial administration of granulocyte-colony stimulating factor (G–CSF)–mobilized autologous peripheral blood (PB)-CD34^+^ cells compared with standard therapy in patients with decompensated cirrhosis type C.

**Methods:**

Patients were randomly assigned (2:1) to the CD34^+^ cell transplant (CD34^+^ cell) or standard-of-care (SOC) group and followed up for 52 weeks. The primary endpoints were the non-progression rate of Child-Pugh (CP) scores at 24 weeks post-enrollment and the safety of the protocol treatment.

**Results:**

Fourteen patients (CD34^+^ cell group: 10; SOC group: 4) were enrolled. CP scores at 24 weeks had a non-progression rate of 90% in the CD34^+^ cell group and 100% in the SOC group, with no significant difference between groups. Importantly, 4 out of 10 patients in the CD34^+^ cell group exhibited an improvement from decompensated to compensated cirrhosis, whereas all patients in the SOC group remained in decompensated cirrhosis. With regard to secondary endpoints, a trend toward increased serum albumin levels in the CD34^+^ cell group was noted. Serious adverse events (SAEs) occurred in three patients in the CD34^+^ cell group and in one patient in the SOC group. No causal relationship was observed between all SAEs and G-CSF, leukapheresis, or cell transplantation in the CD34^+^ cell group. No patients died and no hepatocellular carcinoma occurred within the study period.

**Conclusions:**

PB-CD34^+^ cell infusion therapy may have the potential to circumvent the decompensated stage of cirrhosis, thus avoiding the need for liver transplantation.

## Highlights

• CD34^+^ cell therapy can avoid cirrhosis progression and boost liver reserve function.
• Autologous PB-CD34^+^ cell infusion therapy can circumvent decompensated cirrhosis.
• Autologous PB-CD34^+^ cell infusion therapy can help prevent liver transplantation.

## Graphical abstract

**Figure:**
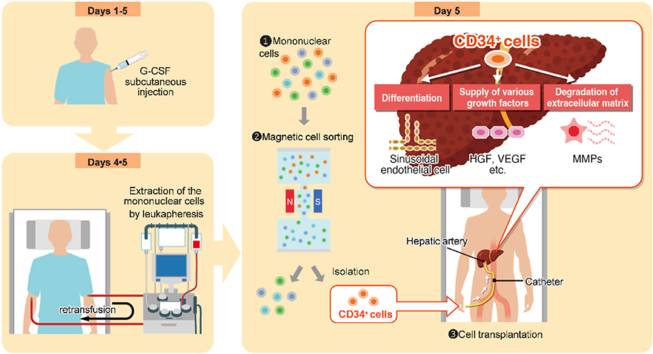

## 1 Introduction

Approximately 400,000 individuals in Japan have cirrhosis, with hepatitis C virus (HCV) infection being the predominant cause [1]. Direct-acting antiviral (DAA) treatment became available in Japan for patients with chronic hepatitis type C and compensated cirrhosis type C in 2014 and has achieved over 95% viral clearance. DAA treatment (sofosbuvir/velpatasvir [SOF/VEL]) for decompensated cirrhosis (DC) became available in Japan in 2019, providing antiviral treatment regardless of disease stage [2]. A phase III study of SOF/VEL in patients with HCV-induced DC reported a 92% sustained virological response (SVR) rate 12 weeks after completion of SOF/VEL treatment (SVR12). Concerning liver functional reserve, the study also reported that 26% of patients in Child–Pugh (CP) Class B (CP–B) at baseline showed improvement in CP class, whereas the remaining 74% showed no improvement, and 2% deteriorated [3]. Among patients who achieved SVR12, 50% of those with CP-B or CP-C exhibited an improvement in CP class pre-SOF/VEL treatment, while 41.4% progressed to CP-A 48 weeks post-treatment [4]. Therefore, most patients could not recover from DC even after SOF/VEL treatment. More effective treatment options for DC after viral elimination are needed.

Cirrhosis treatment aims to improve liver functional reserve and inhibit hepatocellular carcinoma (HCC). Despite achieving SVR with antivirals, studies show that HCV elimination did not directly result in HCC suppression, with 0.9–4.2% of patients developing cancer over a period of 3–8 years [5,6].

Recently, regenerative medicine research using bone marrow (BM) or peripheral blood (PB) hematopoietic/endothelial and mesenchymal stem cells has entered the clinical application stage. Clinical trials are examining the value of stem, cultured, and differentiated non-parenchymal cells for regenerative therapy in liver cirrhosis since they have been shown to contribute to organ function improvements through their antifibrotic effects [[7], [8], [9]]. BM-derived endothelial progenitor cells (EPCs) were found to be present in the mononuclear cell components of human PB and abundant in the CD34^+^ cell fraction [10]. These cells can selectively migrate to sites damaged by inflammation or ischemia and contribute to vasculogenesis in adults. EPCs also contribute to angiogenesis via the paracrine effect [[11], [12], [13], [14], [15]]. Clinical trials worldwide have examined the value of CD34^+^ cells and demonstrated their safety and efficacy for ischemic diseases [[16], [17], [18], [19]].

Using a preclinical murine model of chronic liver injury, we previously observed that engrafted CD34^+^ cells promoted intrahepatic blood vessel formation. We also observed liver fibrosis improvement and tissue regeneration due to the supply of various growth factors secreted from the transplanted cells to the surrounding tissue [20]. Subsequently, we conducted a clinical study of liver regeneration therapy involving the transhepatic arterial administration of autologous PB-CD34^+^ cells in patients with DC [21]. Although the previous study was single-armed and involved few patients without specifying the cause of their cirrhosis, we observed increased hepatic blood flow, improved quality of life (QOL), and significant increases in serum albumin levels in the middle- and high-dose transplant groups compared with the pre-treatment levels. The reference data showed a trend toward worsening of the CP and Model of End-stage Liver Disease (MELD) scores for the historical control group, whereas the transplant group exhibited delayed disease progression, suggesting the efficacy of CD34^+^ cell therapy in preventing cirrhosis progression and improving liver reserve function.

Therefore, this multicenter, open-label, exploratory randomized trial aimed to compare the efficacy and safety of liver regeneration therapy involving the transhepatic arterial administration of granulocyte-colony stimulating factor (G–CSF)–mobilized autologous PB-CD34^+^ cells with standard therapy in patients with DC type C.

## 2 Methods

### 2.1 Study design

This multicenter, prospective, open-label, randomized, controlled trial was conducted in accordance with the Act on Securing Safety of Regenerative Medicine in Japan and the principle of the Declaration of Helsinki. The Specially Certified Committee for Regenerative Medicine (Type 2) approved the study protocol (approval number: SKRM-2-002). This clinical trial was registered in the University Hospital Medical Information Network (no.: UMIN000028965) and Japan Registry of Clinical Trials (no.: jRCTb070190052).

### 2.2 Consent, registration, and assignment

Principal investigators or sub-investigators obtained written informed consent after delivering a comprehensive explanation to patients meeting the inclusion/exclusion criteria. Patients were enrolled and randomly assigned to the CD34^+^ cell transplantation (CD34^+^ cell) and standard-of-care (SOC) groups at a ratio of 2:1 after confirming eligibility. Adjustment factors for allocation were the institution of care and CP score (Class B or C) during the screening period.

### 2.3 Enrollment criteria

The inclusion criteria were: (1) HCV-induced cirrhosis, (2) age 20–80 years at the time of consent, and (3) CP score of ≥7 at two measurement points >90 days apart (for patients treated with DAA, the CP score was determined at two measurement points more than 12 weeks after the completion of DAA treatment if it was not completed following the last dose). Supplementary Table S1 presents the exclusion criteria used to exclude patients at elevated risk for G-CSF treatment, leukapheresis, or use of CD34^+^ cells and to permit optimal evaluation of efficacy.

### 2.4 Treatment and procedures

The protocol treatment was administered according to group allocation. G-CSF (Filgrastim; Gran, Kyowa Hakko Kirin, Japan) administration in the CD34^+^ cell group was initiated within 2 weeks after registration.

Patients assigned to the cell transplant group underwent CD34^+^ cell transplant therapy and standard therapy. G-CSF was subcutaneously administered at a dose of 10 μg/kg per day in these patients for 5 consecutive days to mobilize the BM-derived EPCs and was subsequently discontinued if the white blood cell (WBC) count was ≥75,000/μL. Leukapheresis was performed using a blood component separator (Spectra Optia, Terumo BCT, Tokyo, Japan or COMTEC, Fresenius Kabi Japan Co., Ltd., Tokyo, Japan) on days 4 and 5 after initiating G-CSF administration. Leukapheresis products were diluted to a concentration of <2 × 10^8^ cells/mL using autologous plasma and stored at 4–8 °C until CD34^+^ cells were isolated. Next, CD34^+^ cells were isolated on day 5 after initiating G-CSF administration using a CliniMACS® CD34 Reagent System (Milteny Biotec, Bergisch Gladbach, Germany), CD34 reagent, phosphate-buffered saline/ethylenediaminetetraacetic acid buffer, and tubing set (Miltenyi Biotec, Bergisch Gladbach, Germany). The isolated CD34^+^ cells’ purity and viability were quantified through flow cytometry (FCM) using the Stem Cell Enumeration kit (BD Biosciences, Franklin Lakes, NJ, USA) or Stem-Kit (Beckman Coulter, Mississauga, ON, Canada) according to the International Society of Hematotherapy and Graft Engineering guidelines [[22], [23], [24]].

The recovery of CD34^+^ cells was defined using the following formula: recovery of CD34^+^ cells=(number of CD34^+^ CD45^dim^ cells after cell isolation)/(number of CD34^+^ CD45^dim^ cells before cell isolation).

Femoral artery angiography was performed, and the autologous CD34^+^ cells were infused into the common hepatic artery using a microcatheter (BOBSLED ALLROUNDER, 2.0/2.3Fr., Piolax Medical, Kanagawa, Japan; Sniper®2 MHX-HighFlow, 2.7/2.9Fr., Terumo Clinical Supply Co. Ltd, Gifu, Japan; Carry Leōn HighFlow, 2.6/2.8Fr., UTM Co. Ltd, Aichi, Japan; or ASAHI Tellus, 1.9/2.8Fr., Asahi INTECC, Aichi, Japan) [21].

Patients assigned to the SOC group continued treatment that had been initiated pre-enrollment. Although diuretics could be administered and doses could be changed, albumin transfusions were prohibited within 7 days of the scheduled examination date during the study period.

### 2.5 Endpoints

Based on the 100% non-progression rate of CP score 24 weeks post-enrollment with CD34^+^ cell therapy in the previous clinical study, the primary endpoints (1) the non-progression rate of CP score 24 weeks post-enrollment and (2) the safety of the protocol treatment. Secondary endpoints evaluated to further investigate treatment efficacy were: (1) CP score; (2) MELD score; (3) ascites volume based on abdominal ultrasonography (US) and computed tomography (CT); (4) serum albumin, total bilirubin, prothrombin time-international normalized ratio (PT-INR), and total protein; (5) serum hyaluronic acid and type IV collagen; (6) QOL as assessed using the Short Form 36-Item Health Survey, version 2 (SF-36v2); (7) death due to cirrhosis and all-cause mortality; (8) HCC incidence; and (9) cell separation device issue.

An exploratory endpoint included the changes in albumin–bilirubin (ALBI) score from baseline to 4, 8, 12, 24, 36, and 52 weeks post-enrollment [25].

Additional endpoints included the following parameters related to the primary endpoint: improvement rate in CP score by ≥ 1 point in CP class, and from decompensated to compensated cirrhosis based on CP score/class from baseline to 24 weeks post-enrollment.

### 2.6 Ascites volume measurement via abdominal US and CT

Ascites volume on abdominal US and CT were measured following the methods described by Matsumoto et al. [26] and Oriuchi et al. [27], respectively. These measurements were performed at each follow-up visit (weeks 24 and 52).

### 2.7 QOL analysis

Health-related QOL was evaluated at baseline and weeks 24 and 52 using the SF-36v2, which comprises 36 questions encompassing the following 8 health concepts: physical functioning, role-physical, body pain, general health, vitality, role-emotional, social functioning, and mental health [28,29].

### 2.8 Safety evaluation

All adverse events, including severe events, for safety monitoring were collected irrespective of cause. Physical examination outcomes and vital signs, including hematologic and biochemical findings, were collected during the treatment period and at each follow-up visit (weeks 2, 4, 8, 12, 24, 36, and 52). The severity of adverse events was assessed using the National Cancer Institute Common Terminology Criteria for Adverse Events (version 3.0).

### 2.9 Data management

Patient enrollment, data management, and statistical analysis were performed at an independent data center. Monitoring and audits were conducted for system validation and data quality assurance.

### 2.10 Statistical analysis

According to previous studies, the non-progression rate of CP scores 24 weeks post-enrollment was expected to be 43% and 100% for the SOC and CD34^+^ cell groups, respectively. In total, 21 patients (CD34^+^ cell group: 14 and SOC group: 7) were needed to detect this difference in the therapeutic effect at a one-sided significance level of 5% with 80% power. Allowing for a dropout rate of 10%, the target number of patients was 24 (CD34^+^ cell group: 16 and SOC group: 8).

The safety analysis set (SAF) comprised all patients who received the protocol treatment. Among the SAF, the population excluding “patients who were ineligible post-enrollment” and “those without efficacy data after the protocol treatment” was defined as the full analysis set (FAS) and was used to evaluate the efficacy. Of the FAS, the population excluding patients with transplanted CD34^+^ cell counts <1 × 10^5^ cells/kg was defined as the modified full analysis set (mFAS) and was used to analyze the primary endpoint's sensitivity. Additionally, the population of patients who reached SVR pre-enrollment in this clinical trial was defined as an exploratory subgroup for the subgroup analysis. Furthermore, the CD34^+^ cell separation device performance was evaluated in all patients in the CD34^+^ cell group.

Baseline characteristics were presented as means ± standard deviation (SD) and percentages. Baseline characteristics between groups were compared using the Wilcoxon rank-sum and Fisher's exact tests for continuous and categorical variables, respectively.

The primary endpoint was calculated using Fisher's exact test. The secondary endpoints were also assessed using Fisher's exact test. The time-course of changes in CP, MELD, and ALBI scores and each efficacy parameter after cell therapy were estimated using mixed models for repeated measures.

All reported P-values were two-sided, and statistical significance was set at P < 0.05. Statistical analyses were conducted using SAS software, version 9.4 (SAS Institute, Cary, NC, USA).

## 3 Results

### 3.1 Patients

**Fig. 1:**
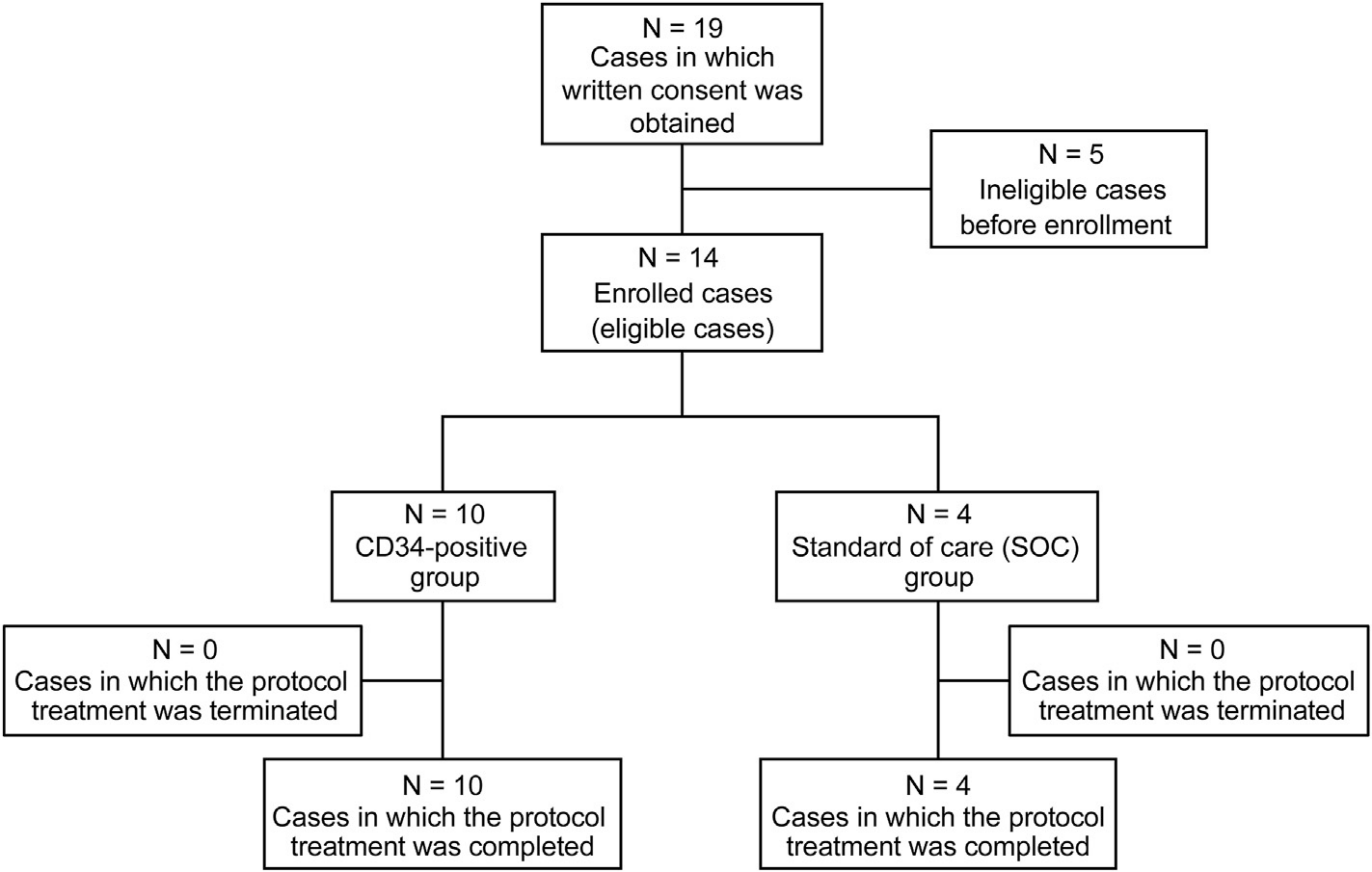
Study design and participant allocation.

Fourteen patients were enrolled between December 2017 and February 2022 and assigned at a 2:1 ratio to the CD34^+^ cell and SOC groups (10 and 4 patients, respectively). Although the registration deadline was extended from September 2019 to February 2022, the target number of patients (n = 24) could not be reached. Ultimately, patient enrollment was terminated in February 2022, and follow-up of the last patient was completed in November 2022 (Fig. 1).

Table 1 presents the patient background characteristics. The CP scores, which reflect the patient's liver functional reserve, were 8.0 ± 1.2 and 8.0 ± 1.4 points in the CD34^+^ cell and SOC groups, respectively. Regarding HCV elimination, two non-SVR and eight SVR patients were enrolled in the CD34^+^ cell group, while two non-SVR and two SVR patients were in the SOC group. The distribution was skewed by smoking status (P = 0.031, Table 1).

**Table 1:** Baseline characteristics.

Characteristics	CD34^+^ cell group	SOC group	P-value
	(N = 10)	(N = 4)	
Age (years)	62.8 ± 9.1	70.8 ± 7.8	0.066
Sex, no. (%)	7/3 (70.0/30.0)	1/3 (25.0/75.0)	0.245
Male/Female			
Child-Pugh class, no.	0/9/1 (0.0/90.0/10.0)	0/3/1 (0.0/75.0/25.0)	0.505
Class A/B/C			
Child-Pugh score (point)	8.0 ± 1.2	8.0 ± 1.4	1.000
MELD score (point)	11.4 ± 2.1	10.5 ± 2.6	0.473
AST (U/L)	41.8 ± 20.4	50.5 ± 17.1	0.357
ALT (U/L)	31.6 ± 18.2	34.5 ± 9.6	0.572
Albumin (g/dL)	3.43 ± 0.42	3.24 ± 0.31	0.545
Total bilirubin (mg/dL)	1.95 ± 0.87	1.80 ± 0.70	0.644
Prothrombin time (%)	68.59 ± 10.99	68.05 ± 14.12	0.745
PT-INR	1.24 ± 0.11	1.24 ± 0.16	0.980
Creatinine (mg/dL)	0.73 ± 0.23	0.64 ± 0.15	0.671
Platelets ( × 10^4^/μL)	10.18 ± 5.40	9.03 ± 1.77	0.571
HCV-RNA, no. (%)	8/2 (80.0/20.0)	2/2 (50.0/50.0)	0.520
Negative/Positive			
Alcohol, no. (%)	2/8 (20.0/80.0)	0/4 (0.0/100.0)	1.000
Almost never drink/Do not drink			
Smoking, no. (%)			0.031
Current smoker	5 (50.0)	0	
Past smoker	3 (30.0)	0	
Non-smoker	2 (20.0)	4 (100.0)	
Number of cigarettes (cigarettes/day)	10.4 ± 8.4	0	–
Years of smoking (years)	35.3 ± 10.4	0	
Brinkman Index	354.6 ± 303.3	0	
History of hepatocellular carcinoma, no. (%)	8/2 (80.0/20.0)	3/1 (75.0/25.0)	1.000
No/Yes			
Diabetes mellitus, no. (%)	3 (30.0)	1 (25.0)	1.000
Hypertension, no. (%)	2 (20.0)	1 (25.0)	1.000
Dyslipidemia, no. (%)	0	0	–
Proliferative retinopathy, no. (%)	0	0	–

Data are presented as mean ± standard deviation (SD). HCV-RNA, hepatitis C virus-RNA; PT-INR, prothrombin time-international normalized ratio; SOC, standard-of-care; MELD, Model of End-stage Liver Disease; ALT, alanine transaminase; AST, aspartate transaminase; no., number.

### 3.2 Mobilization, harvesting, and isolation of CD34^+^ cells

G-CSF administration was not discontinued in any patient since the WBC count never exceeded 75,000/μL during the G-CSF treatment period. The leukapheresis products contained 435.1 ± 194.2 (× 10^8^) and 321.6 ± 247.7 (× 10^8^) cells on leukapheresis days 1 and 2, respectively. The number of CD34^+^ cells in the leukapheresis product was 64.2 ± 30.2 (× 10^6^) and 101.4 ± 52.2 (× 10^6^) cells on leukapheresis days 1 and 2, respectively. FCM analysis showed that the purity, viability, cell count, and recovery of the CD34^+^ cell fraction after magnetic sorting were 74.8 ± 14.2%, 91.5 ± 6.9%, 69.8 ± 45.1 (× 10^6^) cells, and 70.8 ± 18.3%, respectively. The number of transplanted CD34^+^ cells was 58.9 ± 42.6 (× 10^6^) cells/patient and 0.9 ± 0.6 (× 10^6^) cells/kg body weight.

### 3.3 Efficacy evaluation

**Fig. 2:**
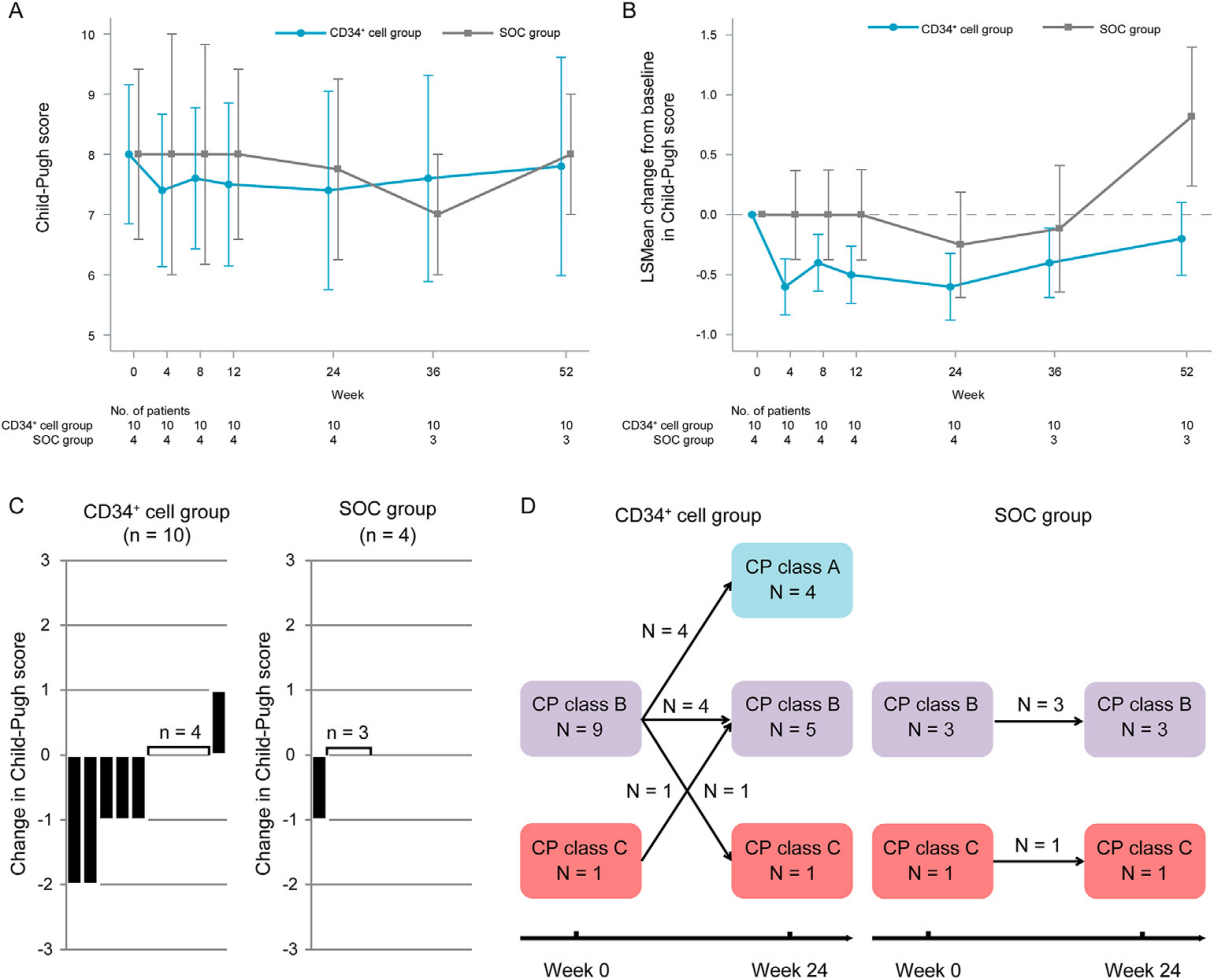
The time-course of changes in Child–Pugh scores and class in the CD34^+^ cell and SOC groups. (A) The time-course of changes in Child–Pugh scores. (B) The time-course of changes in Child–Pugh scores from baseline to each time point. (C) The changes in Child–Pugh scores from baseline to 24 weeks post-enrollment. (D) The distributional changes in Child–Pugh classes from baseline to 24 weeks post-enrollment. Data are presented as (a) mean ± SE or (b) LSMean ± SE. LSMean: Least squares means; SE: standard error; SOC, standard-of-care.

Regarding the primary endpoint, no significant difference was observed in the non-progression rate of CP scores (95% confidence interval [CI]) at 24 weeks post-enrollment in the FAS; 90% (55.5, 99.7) and 100.0% (39.8, 100.0) in the CD34^+^ cell and SOC groups, respectively (P = 1.000). The non-progression rate of CP score (95% CI) at 24 weeks post-enrollment for the mFAS was 100.0% (66.4, 100.0) and 100.0% (39.8, 100.0) for the CD34^+^ cell and SOC groups, respectively, without a significant difference (P-value not calculable). Additionally, the changes in CP score from baseline to each time point were not significantly different between the groups (Fig. 2A and B).

Additional analysis related to the primary endpoint in the CD34^+^ cell group showed CP score and class improvement in 5 of the 10 patients (50%) and improvement from decompensated to compensated cirrhosis in 4 (40%) at 24 weeks post-enrollment. In the SOC group, CP scores improved in one of the four patients (25%); however, CP class did not improve in all patients, and all patients remained in the decompensated stage of cirrhosis at 24 weeks post-enrollment (Fig. 2C and D). Although no statistically significant difference was observed between the groups, improvement rates in CP score, CP class, and reversal from decompensated to compensated cirrhosis tended to be higher in the CD34^+^ cell group than in the SOC group at 24 weeks post-enrollment for the FAS (Tables S2–4). The results were similar for the mFAS (data not shown).

**Fig. 3:**
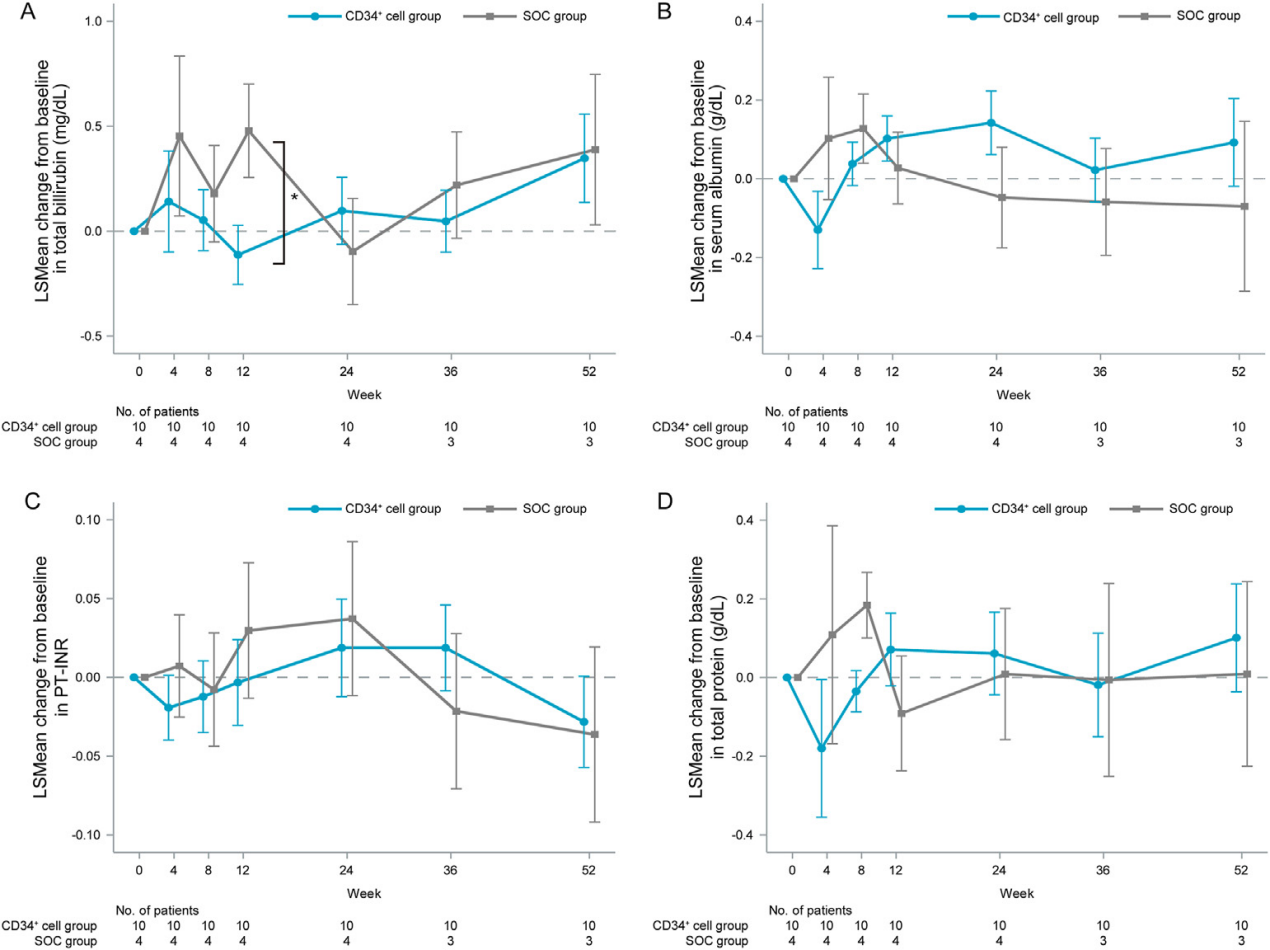
The time-course of changes in liver functional reserve-related parameters in the CD34^+^ cell and SOC groups. The changes in (A) total bilirubin, (B) serum albumin, (C) PT-INR, and (D) total protein from baseline to each time point post-enrollment. Data are presented as LSMean ± SE. LSMean: Least Squares Means; PT-INR, prothrombin time-international normalized ratio; SOC, standard-of-care. ∗P < 0.05 for intergroup comparisons at each point.

For the secondary endpoints, when total bilirubin, serum albumin, PT-INR, and total protein were evaluated over the time-course changes, a significant intergroup difference in the total bilirubin level was observed at 12 weeks post-enrollment (P = 0.046), with higher total bilirubin levels found in the SOC group than in the CD34^+^ cell group (Fig. 3A). Serum albumin levels increased at 24 weeks post-enrollment in the CD34^+^ cell group compared with the SOC group without significant intergroup differences at any point (Fig. 3B). No albumin transfusions were performed in either group. Similarly, PT-INR and total protein did not significantly differ between the groups at any point (Fig. 3C and D).

**Fig. 4:**
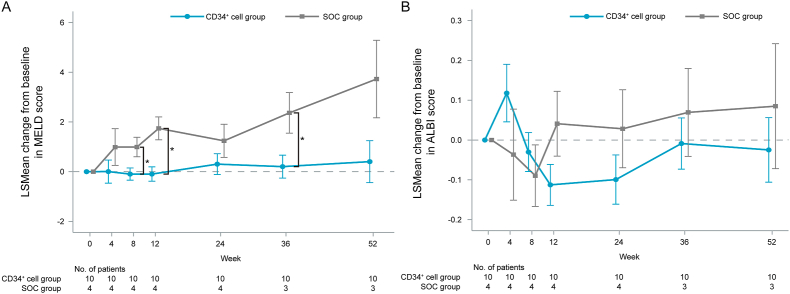
The changes in the (A) Model for End-stage Liver Disease (MELD) and (B) albumin–bilirubin (ALBI) scores from baseline to each time point post-enrollment in the CD34^+^ cell and SOC groups. Data are presented as LSMean ± SE. LSMean: Least Squares Means; SOC, standard-of-care. ∗P < 0.05 for intergroup comparisons at each time point.

Interestingly, significant intergroup differences were found in the MELD score changes at 8, 12, and 36 weeks post-enrollment (P = 0.039, 0.006, 0.039, respectively), with worse scores in the SOC group than in the CD34^+^ cell group (Fig. 4A). None of the patients in either group had the administration or dosage of the diuretic modified.

No significant intergroup differences in ascites volume changes on abdominal US or CT (Figs. S1A and B). The serum hyaluronic acid changes showed a trend toward improvement in both groups, however there was no significant difference between groups at any point (Fig. S2A). The type IV collagen level changes showed an increasing trend in the SOC group but was not significantly different between groups at any point (Fig. S2B).

**Fig. 5:**
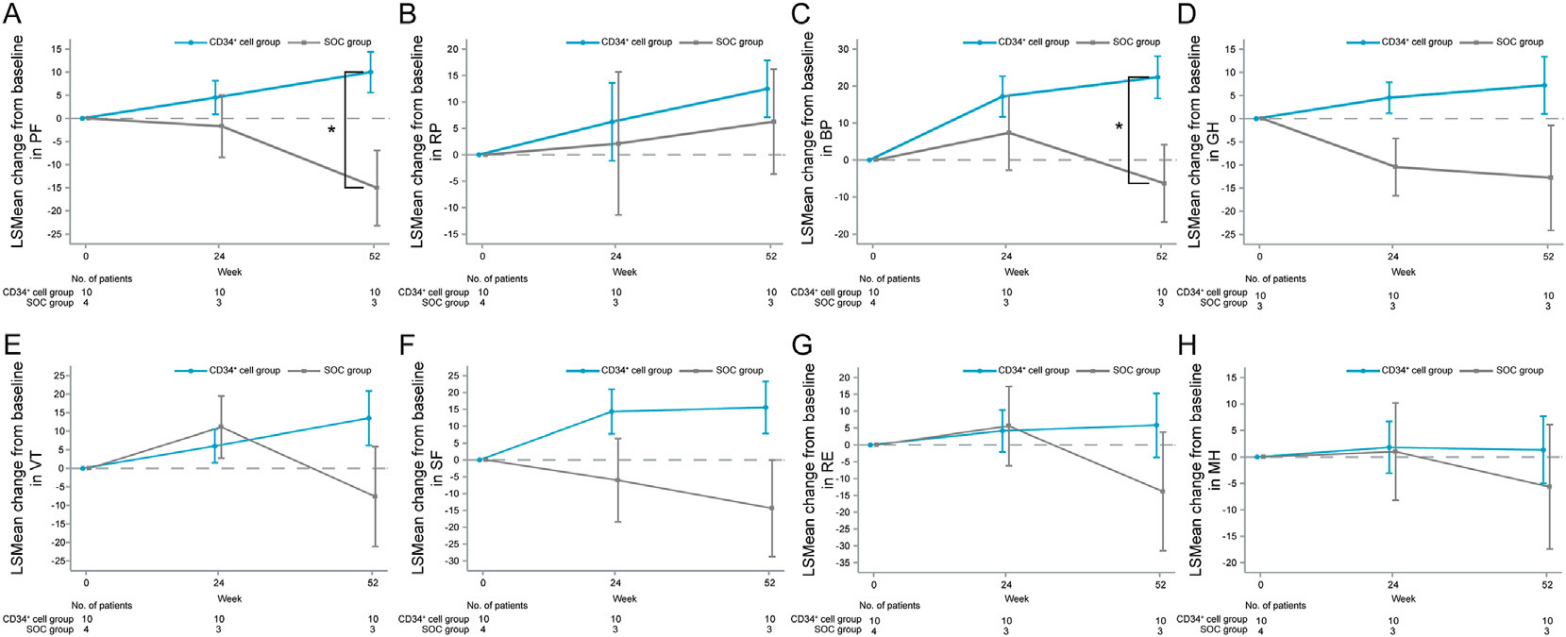
The time-course of changes from baseline to 24 and 52 weeks post-enrollment in SF-36v2 subscale scores (0–100) of (A) physical functioning (PF), (B) role-physical (RP), (C) body pain (BP), (D) general health (GH), (E) vitality (VT), (F) social functioning (SF), (G) role-emotional (RE), and (H) mental health (MH) in the CD34^+^ cell and SOC groups. Data are presented as LSMean ± SE. LSMean: Least Squares Means; SF-36v2, Short Form 36-Item Health Survey, version 2. ∗P < 0.05 for intergroup comparisons at each time point.

In assessing changes in QOL ratings, significant intergroup differences in subscale scores for physical function (PF) and body pain (BP) were observed at 52 weeks post-enrollment (P = 0.022, P = 0.043), with the CD34^+^ cell group exhibiting an increase in QOL (Fig. 5A–H). Similarly, significant intergroup differences in PF and BP scores were observed at 52 weeks post-enrollment (P = 0.022, P = 0.043; Figs. S3A–H). Significant intergroup differences were also observed in the physical component summary (PCS) of the two-component summary score at 52 weeks post-enrollment (1995 US National Survey Data) (P = 0.040; Figs. S4A and B).

### 3.4 Safety evaluation

Mild adverse events, particularly G–CSF– or leukapheresis-related events, were frequent; however, all mild adverse events were transient and improved without sequelae (Table S5). SAEs occurred in three (vascular stent occlusion: one, ascites: one, and hepatic encephalopathy: one) and one (hepatic encephalopathy, pleural effusion, and pleurisy) patients in the CD34^+^ cell and SOC groups, respectively. However, no causal associations with G-CSF, leukapheresis, or cell transplantation were found in the CD34^+^ cell group. Additionally, nine non-SAEs related to cell transplantation occurred. No adverse events were associated with the use of the cell separation device. Two cell separation device complications occurred, and no patients died or developed HCC during the study period. Furthermore, no appearance of antibodies to mouse-derived proteins was also observed (data not shown).

### 3.5 An exploratory endpoint

The time-course changes in ALBI scores from baseline did not significantly differ between the groups at any point (Fig. 4B).

### 3.6 Subgroup analysis in patients with SVR

Since a significant intergroup difference in the non-progression rate of CP scores at 24 weeks post-enrollment could not be found in the population included in the efficacy analysis, the same primary endpoint analysis was performed in patients with SVR (CD34^+^ cell group: eight, SOC group: two). The frequency of subjects fulfilling the primary endpoint in patients with SVR was 100% in both groups. Although the time-course changes in the CP scores from baseline were also evaluated, no significant intergroup differences were found at any point (Fig. S5A).

An exploratory analysis was performed on patients with SVR to examine the change in ALBI scores from baseline to 24 weeks post-enrollment to assess the efficacy regarding liver functional reserve. The mean change was −0.13 ± 0.07 and 0.10 ± 0.14 in the CD34^+^ cell and SOC groups, respectively (P = 0.179, Table S6). The time-course changes in ALBI scores were also evaluated, although no significant intergroup differences were observed at any point (Fig. S5B).

Additionally, the improvement rate in CP scores by ≥ 1 point at 24 weeks post-enrollment was examined in patients with SVR, which was 62.5% and 50% in the CD34^+^ cell and SOC groups, respectively. Moreover, four of the eight patients (50%) in the CD34^+^ cell group exhibited an improvement from decompensated to compensated cirrhosis, while all patients in the SOC group remained in the decompensated stage of cirrhosis (Fig. S5C). Although no significant difference was found between the groups, the improvement rates in CP score, CP class, and the reversal from decompensated to compensated cirrhosis tended to be higher in the CD34^+^ cell group than in the SOC group (Tables S7–9).

## 4 Discussion

Liver transplantation is the only established treatment for advanced DC; however, its use remains limited due to the shortage of donors and the patient's surgical tolerance. Ideally, progression to liver cirrhosis must be prevented using appropriate measures against the underlying disease in its early stages. However, numerous studies are currently examining the potential of novel therapies to resolve the cirrhotic state and restore the liver's original regenerative ability [30].

No clinical trial guidelines or recommendations existed to standardize the design and definition of endpoints for patients with DC when this clinical trial was begun in 2017 [21]. Therefore, the design and endpoints of this clinical trial were based on the results of a previous study [21]. Recently, the LiverHope Consortium Expert Panel recommended that clinical trials involving patients with DC and proof-of-concept studies should include robust surrogate markers closely associated with survival (i.e., changes in CP or MELD scores) as the primary endpoint and biomarkers of disease progression or impairment as surrogate endpoints [31]. Compared to drug-based therapy, cell therapy generally has various mechanisms of action; therefore, considering appropriate endpoints for each cell type is necessary. In our basic studies, transplanted EPCs have been found to differentiate directly into endothelial cells and secrete cytokines/growth factors that induce angiogenesis, anti-inflammation, and anti-fibrosis [20,32]. We believe that EPCs can improve clinical and functional parameters over a specific time-course through such physiological processes. Therefore, this study evaluated several parameters, including the CP score, MELD score, the five components of the CP score, and QOL, which represent clinically important indicators to explore this critical issue.

During this study period, DAA treatment for DC type C became available in Japan. Therefore, patients enrolled after 2020 were all those with DC regardless of achieving SVR after antiviral therapy with DAA. Based on the results of our previous clinical trial [21], this prospective exploratory clinical trial was conducted using the non-progression rate of CP scores at 24 weeks post-enrollment as the primary endpoint compared with standard treatment; however, no significant difference in CP scores was observed between the groups. Possible reasons for the failure to demonstrate primary endpoint superiority in this study were: 1) the non-progression rate of CP scores in the SOC group was higher than previously estimated (64% in the previous study) due to patients with SVR inclusion in the enrollment, and 2) the power of detection was reduced because of the limited number of registered patients. Nevertheless, a trend toward increased serum albumin levels with CD34^+^ cell transplantation was observed, similar to the previous clinical trial's results [21]. Moreover, significant differences were found in the change in MELD scores at 8, 12, and 36 weeks post-enrollment. The MELD score is calculated based on total bilirubin, creatinine, and PT-INR values. In this study, total bilirubin levels tended to be higher in the SOC group than in the CD34^+^ cell group, with a significant difference especially at 8 weeks post-enrollment. Creatinine levels tended to be higher in the SOC group than in the CD34^+^ cell group after 8 weeks post-enrollment (no significant difference) (Fig. S6). On the other hand, no particular trend can be observed in PT-INR levels between the groups. Overall, these results suggest that the changes in MELD scores at 8, 12, and 36 weeks post-enrollment were significantly improved in the CD34^+^ cell group compared to the SOC group, indicating that the CD34^+^ cell group was superior to standard treatment. Furthermore, a subgroup analysis of patients with SVR showed a marked improvement in CP scores in the CD34^+^ cell group, with four of the eight patients improving from decompensated to compensated cirrhosis.

Little is known about the long-term effect of DAA treatment on liver function after achieving SVR in patients with DC. Krassenburg et al. [2] reported that in patients with SVR following DAA treatment (median: 28 months post-treatment), patients who improved to CP-A and those who remained at CP-B/C showed a reduced risk of clinical disease progression and no improvement in clinical prognosis, respectively. A long-term follow-up study (median: 4 years post-treatment) by Verna et al. [33] also reported that patients with SVR after DAA treatment exhibited minimal mean changes in MELD scores (−0.30 points), total bilirubin (+0.23 mg/dL), and albumin (+0.36 g/dL) relative to baseline.

Since no intergroup difference was found in the non-progression rate of CP scores, a parameter of liver functional reserve in this study, an exploratory analysis was conducted in patients with SVR to determine a new parameter of liver functional reserve. We noted that serum albumin levels showed an increasing trend in the CD34^+^ cell group, and total bilirubin levels showed significant intergroup differences at 12 weeks post-enrollment. Therefore, we examined a new parameter, the ALBI score, which evaluates liver functional reserve using a scoring system based on serum albumin and total bilirubin levels. The ALBI score has recently been used to evaluate liver functional reserve in selecting treatment for HCC [34]. Although no significant difference was found in the change in ALBI scores between the groups at 24 weeks post-enrollment in this study, we believe this parameter is superior to the CP score because it can sensitively evaluate liver functional reserve. In an additional analysis of patients with SVR, the improvement rate in CP scores by ≥ 1 point at 12 and 24 weeks post-enrollment was 62.5% and 62.5% in the CD34^+^ cell group and 0% and 50% in the SOC group, respectively. During the previous investigation, all eligible patients were patients without SVR; however, since DAA can presently eliminate the HCV that causes liver damage, CD34^+^ cell therapy is expected to improve CP scores. Therefore, improvement rate in CP scores by ≥ 1 point may be a more appropriate endpoint in the next phase of clinical trials rather than the non-progression rate of CP scores to be evaluated at 12–24 weeks following CD34^+^ cell therapy. In the next clinical trial, we would like to include only SVR patients after antiviral therapy, and expand to include patients with metabolic dysfunction-associated steatohepatitis (MASH)-related cirrhosis, in which the number of patients is growing [1].

This study had some limitations, including the failure to reach the target number of patients. Therefore, if the efficacy of CD34^+^ cell therapy can be established in the next phase of the clinical trial, it would be interesting to subsequently compare its potential with other stem cell therapies. Lastly, further clinical trials are necessary to ascertain the best approach to evaluate clinical improvement in liver functional reserve.

## 5 Conclusions

The results of this trial provide important insight into the safety, feasibility, and potential efficacy of G–CSF–mobilized autologous CD34^+^ cell therapy for patients with DC type C. Additionally, autologous PB-CD34^+^ cell infusion therapy may be a beneficial treatment for DC, with the potential to reverse it into compensated cirrhosis, thereby preventing the need for liver transplantation.

## Author contributions

T.N.: principal investigator, conception and design, provision of study material or patients, collection and assembly of data, manuscript writing.

A.M., H.K., H.S.: conception and design, data analyses and interpretation.

M.Kako, H.E., M.Kaibori: principal investigator, provision of study material or patients, collection and/or assembly of data.

Y.Fujita: conception and design, data interpretation, and technical advisor on cell isolation.

Y.Fujimori, K.K., T.Takashima, H.U., H.Y., K.Y.: provision of study material or patients, collection and/or assembly of data.

K.F., T.H., H.I., Y.K., K.N., T.O., T.Tsukiyama, K.Yamahara, K.Yamakado, S.Y.: technical advisors on cell isolation and transplantation.

K.T., T.I.: data analyses as statisticians.

A.K.: conception and design, data analyses, interpretation, and final approval of the manuscript.

S.N., S.K., T.Torimura, T.K.: conception and design, interpretation and final approval of the manuscript.

## Funding

This research was supported by the Research Project for Practical Application of Regenerative Medicine from the Japan Agency for Medical Research and Development (AMED, Tokyo, Japan) under Grant Number JP20bk0104106.

## Data availability

The datasets in this study are available upon request from the corresponding author.

## Declaration of competing interest

None.
