# Supplementary material for: Hepatic arterial infusion of autologous CD34+ cells for hepatitis C virus-related decompensated cirrhosis: A multicenter, open-label, exploratory randomized controlled trial
Source: Regen Ther. 2024 May 4;27:455–63. doi: 10.1016/j.reth.2024.04.018 (PMC11087913; doi:10.1016/j.reth.2024.04.018)
Supplement: Multimedia component 1 [file mmc1.docx]

**Supplementary Material**

**Hepatic arterial infusion of autologous CD34^+^ cells for hepatitis C virus-related decompensated cirrhosis: a multicenter, open-label, exploratory randomized, controlled trial**

**Authors**

Toru Nakamura^a,b*^, Atsutaka Masuda^a,b^, Makoto Kako^c^, Hirayuki Enomoto^d^, Masaki Kaibori^e^, Yasuyuki Fujita^f^, Kyoko Tanizawa^f^, Tetsuya Ioji^f^, Yoshihiro Fujimori^g^, Kei Fukami^h^, Takuma Hazama^h^, Hideki Iwamoto^a,b^, Yasukazu Kako^i^, Kaoru Kobayashi^i,j^, Hironori Koga^a,b^, Koji Nagafuji^k^, Takayasu Ohtake^l^, Hiroyuki Suzuki^a,b^, Tomoyuki Takashima^d^, Toshitaka Tsukiyama^m^, Haruki Uojima^c,n^, Kenichi Yamahara^o^, Koichiro Yamakado^i^, Hidekazu Yamamoto^e^, Kazunori Yoh^d,p^, Satoshi Yoshihara^g^, Atsuhiko Kawamoto^f^, Shuhei Nishiguchi^d,q^, Shuzo Kobayashi^r^, Takuji Torimura^a,s^, and Takumi Kawaguchi^a^

**Affiliations**

^a^Division of Gastroenterology, Department of Medicine, Kurume University School of Medicine, Kurume, Fukuoka, 8300011, Japan

^b^Liver Cancer Research Division, Research Center for Innovative Cancer Therapy, Kurume University, Kurume, Fukuoka, 8300011, Japan

^c^Gastroenterology Medicine Center, Shonan Kamakura General Hospital, Kamakura, Kanagawa, 2478533, Japan

^d^Division of Hepatobiliary and Pancreatic Diseases, Department of Gastroenterology, Hyogo Medical University, Nishinomiya, Hyogo, 6638501, Japan

^e^Department of Surgery, Kansai Medical University, 2-5-1 Shinmachi, Hirakata, 5731191, Japan

^f^Translational Research Center for Medical Innovation, Foundation for Biomedical Research and Innovation at Kobe, Kobe, Hyogo, 6500047, Japan

^g^Department of Transfusion Medicine and Cellular Therapy, Hyogo Medical University, Nishinomiya, Hyogo, 6638501, Japan

^h^Division of Nephrology, Department of Medicine, Kurume University School of Medicine, Kurume, Fukuoka, 8300011, Japan

^i^Department of Radiology, Hyogo Medical University, Nishinomiya, Hyogo, 6638501, Japan

^j^Department of Radiology, Kawanishi City Medical Center, Kawanishi, 6660017, Japan

^k^Division of Hematology and Oncology, Department of Medicine, Kurume University School of Medicine, Kurume, Fukuoka, 8300011, Japan

^l^Department of Regenerative Medicine, The Center for Cell Therapy & Regenerative Medicine, Shonan Kamakura General Hospital, Kamakura, Kanagawa, 2478533, Japan

^m^Department of Radiology and Interventional Radiology, Shonan Kamakura General Hospital, Kamakura, Kanagawa, 2478533, Japan

^n^Department of Genome Medical Sciences Project, Research Institute, National Center for Global Health and Medicine, Ichikawa, Chiba, 2728516, Japan

^o^Laboratory of Molecular and Cellular Therapy, Institute for Advanced Medical Sciences, Hyogo Medical University, Nishinomiya, Hyogo, 6638501, Japan

^p^Yoh Digestive Clinic, Wakayama, 6408269, Japan

^q^Department of Gastroenterology, Kano General Hospital, Osaka, Japan, 5310041, Japan

^r^Department of Kidney Disease and Transplant Center, Shonan Kamakura General Hospital, Kamakura, Kanagawa, 2478533, Japan

^s^Department of Gastroenterology, Omuta City Hospital, Omuta, 8368567, Japan

**Table of Contents**

1. Supplementary Figure S1
2. Supplementary Figure S2
3. Supplementary Figure S3
4. Supplementary Figure S4
5. Supplementary Figure S5
6. Supplementary Figure S6
7. Supplementary Table S1
8. Supplementary Table S2
9. Supplementary Table S3
10. Supplementary Table S4
11. Supplementary Table S5
12. Supplementary Table S6
13. Supplementary Table S7
14. Supplementary Table S8
15. Supplementary Table S9

# Supplementary Figure


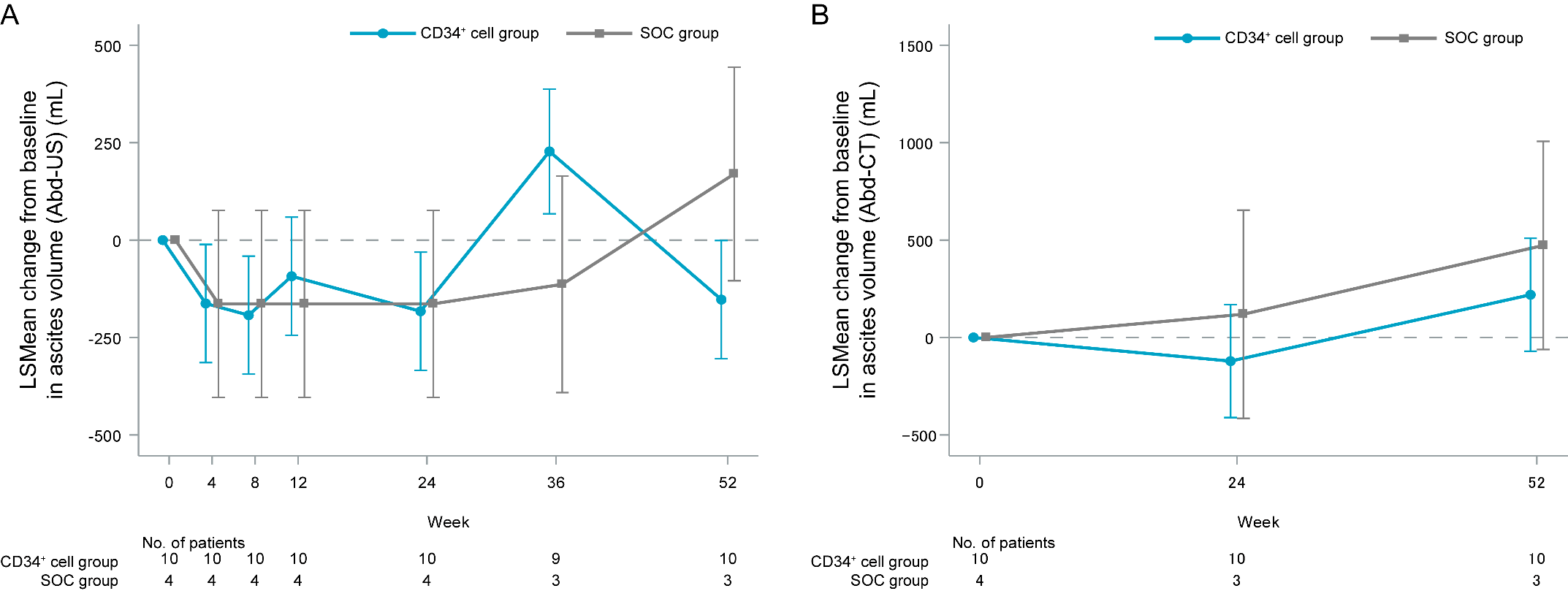


## Supplementary Figure S1. The time-course changes in ascites volume by (A) abdominal ultrasound (Abd-US) and (B) abdominal computed tomography (Abd-CT) from baseline to each time point post-enrollment in the CD34^+^ cell and SOC groups. Data are presented as LSMean ± SE. LSMean: Least Squares Means.

**
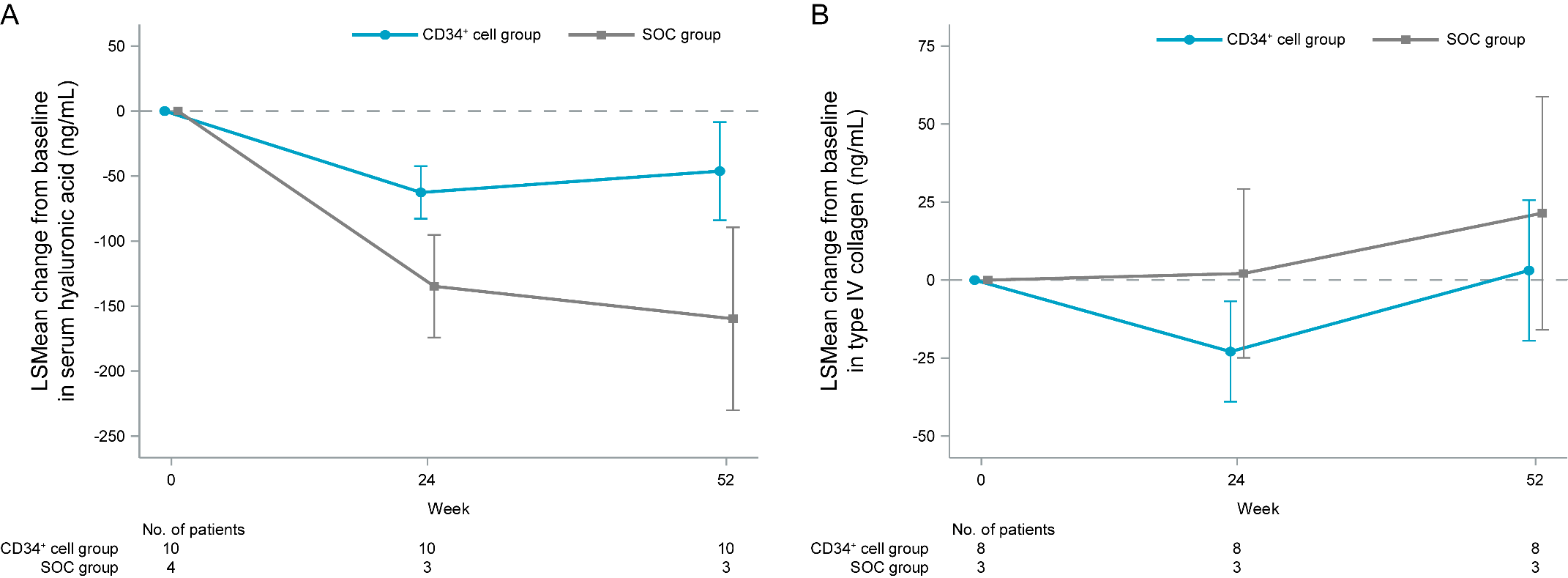
**

**Supplementary Figure S2.** The time-course changes in (A) serum hyaluronic acid and (B) type Ⅳ collagen from baseline to 24 and 52 weeks post-enrollment in the CD34^+^ cell and SOC groups. Data are presented as LSMean ± SE. LSMean: Least Squares Means.


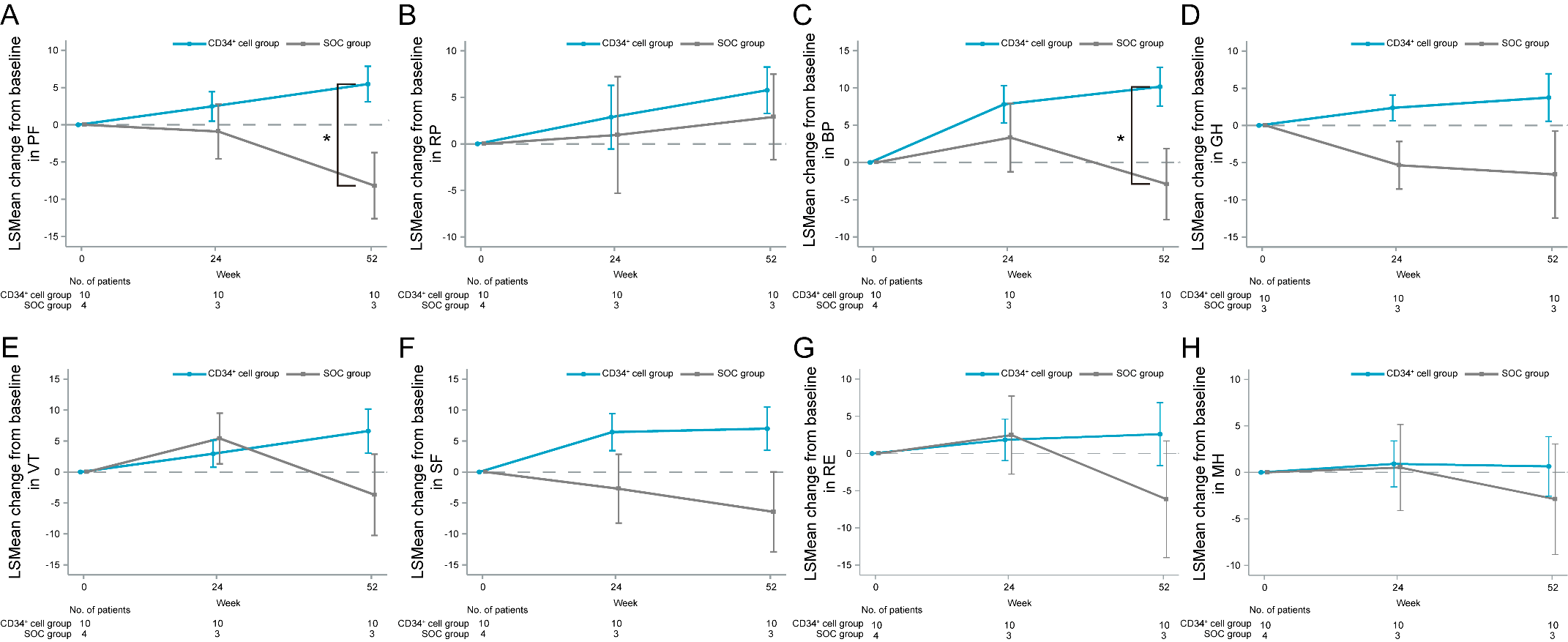


**Supplementary Figure S3.** The time-course changes from baseline to 24 and 52 weeks post-enrollment in SF-36v2 subscale (NBS score) of (A) physical functioning (PF), (B) role-physical (RP), (C) body pain (BP), (D) general health (GH), (E) vitality (VT), (F) social functioning (SF), (G) role-emotional (RE), and (H) mental health (MH) in the CD34^+^ cell and SOC groups. Data are presented as LSMean ± SE. LSMean: Least Squares Means; SF-36v2, Short Form 36-Item Health Survey, version 2. *P<0.05 for intergroup comparisons at each point.


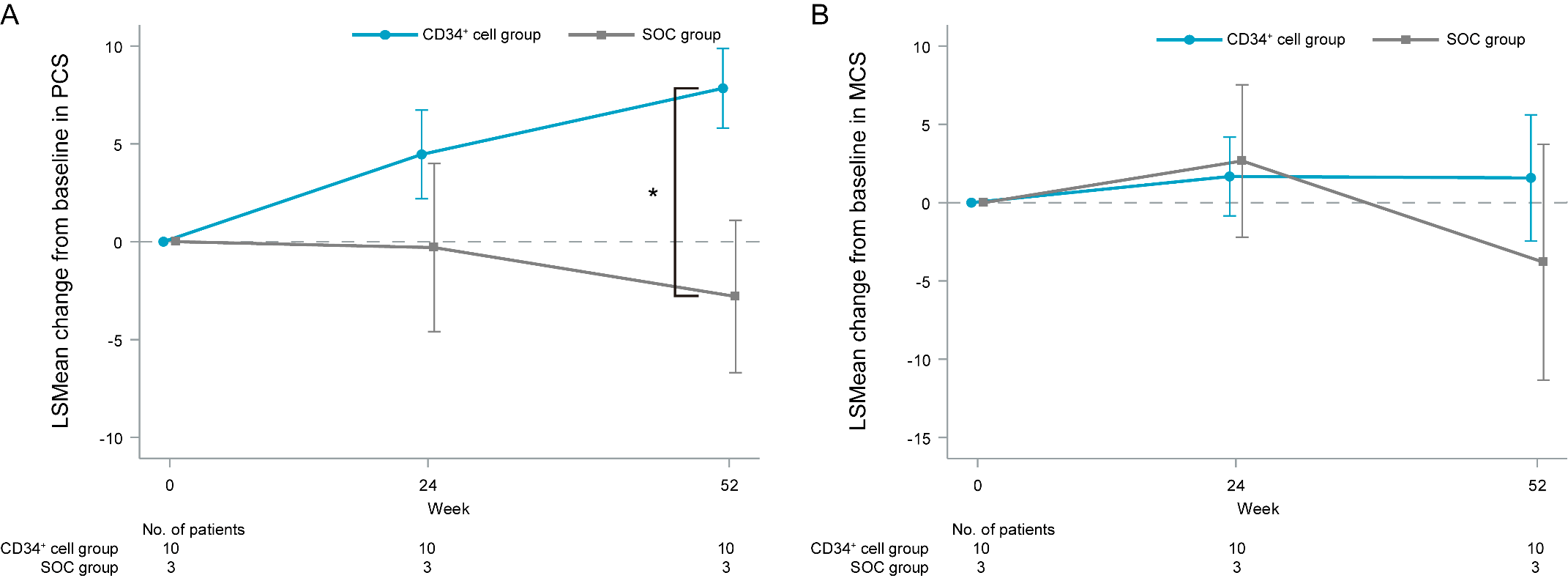


**Supplementary Figure S4.** The time-course changes from baseline to 24 and 52 weeks post-enrollment in SF-36v2 component summary scores (1995 US National Survey Data) of (A) physical component summary (PCS) and (B) mental component summary (MCS) in the CD34^+^ cell and SOC groups. Data are presented as LSMean ± SE. LSMean: Least Squares Means; SF-36v2, Short Form 36-Item Health Survey, version 2. *P<0.05 for intergroup comparisons at each point.


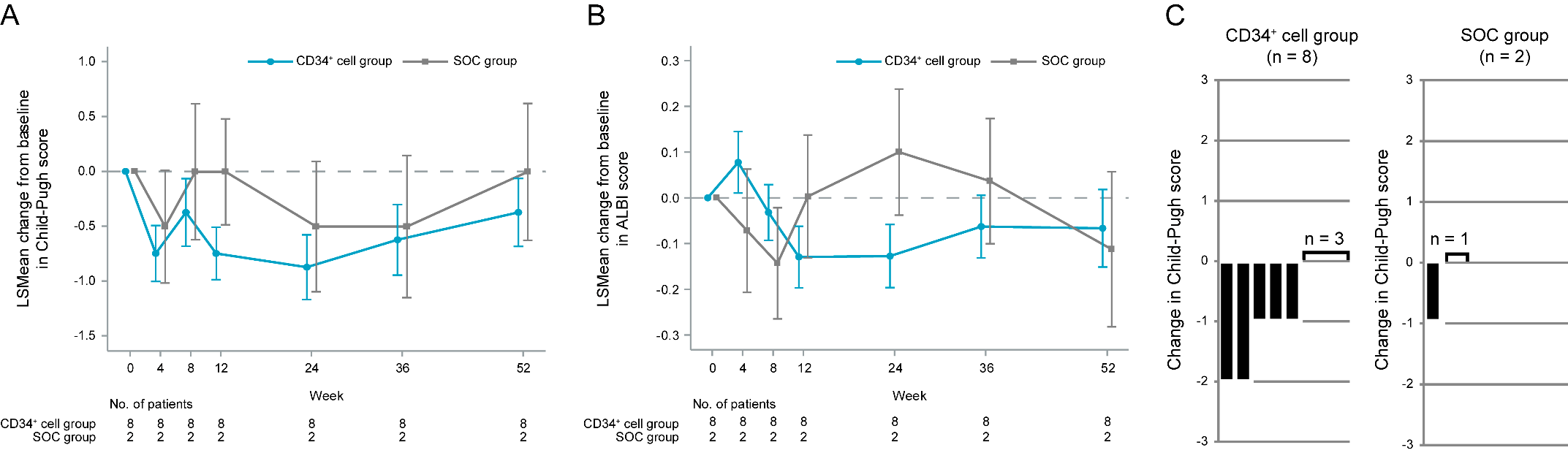


**Supplementary Figure S5.** Subgroup analysis in patients showing sustained virological response. The changes in (A) Child–Pugh scores and (B) albumin–bilirubin (ALBI) scores from baseline to each time point post-enrollment in the CD34^+^ cell and SOC groups. (C) The distributional changes in Child–Pugh score from baseline to 24 weeks post-enrollment in the CD34^+^ cell and SOC groups. Data are presented as LSMean ± SE. LSMean: Least Squares Means.

## Supplementary Figure S6. The changes in creatinine from baseline to each time point post-enrollment in the CD34^+^ cell and SOC groups. Data are presented as LSMean ± SE. LSMean: Least Squares Means.

# Supplementary Table

# Supplementary Table S1. Exclusion criteria.

(1) Liver cirrhosis caused by conditions other than hepatitis C virus (HCV) infection or cryptogenic cirrhosis.

(2) Positive for antibodies against HBs antigen, HIV antibody, HTLV-1 antibody, syphilis serology, or Positive HBs antigen, HIV antibody, HTLV-1 antibody, syphilis serology, or high anti-HBc titer (>10.00 S/CO by CLIA method).

(3) Heavy alcohol consumption or improvements to a Child–Pugh (CP) score of ≤6 after at least 6 months of alcohol abstinence in previously heavy drinkers.

(4) Malignant tumors or history of malignancy within 5 years. However, patients with intraepithelial cancer, such as colorectal intramucosal cancer, were not excluded if there was no recurrence during the screening period. Patients with a history of HCC were also included if there was no recurrence on diagnostic imaging and alpha-fetoprotein (AFP) and protein induced by vitamin K deficiency or antagonist II (PIVKA-II) levels were normal at screening. Patients with elevated AFP or PIVKA-II levels were not excluded if diagnostic imaging performed since the completion of HCC treatment and during the screening period confirmed that there was no recurrence of HCC for at least 3 months.

(5) Total bilirubin level >5.0 mg/dL.

(6) Prothrombin time <30%.

(7) Serum creatinine >2.0 mg/dL.

(8) Hemoglobin <8.0 g/dL.

(9) Platelet count ≤20, 000/μL.

(10) Splenomegaly with a long diameter of >15 cm on abdominal CT.

(11) Gastrointestinal bleeding or risk of gastrointestinal bleeding.

(12) Portal vein thrombosis (although patients with thrombus confined to a part of the portal vein, such as mural thrombus, which is judged not to affect the disease, were not excluded).

(13) Current or history of interstitial pneumonia

(14) Leukemia, myeloproliferative disorders, myelodysplastic syndrome, or sickle cell disease.

(15) Autoimmune disease.

(16) Less than 3 months since the onset of unstable angina, myocardial infarction, or stroke or less than 3 months after coronary artery stent, carotid artery stent, or intracranial artery stent implantation.

(17) Diabetic proliferating retinopathy (new Fukuda classification BII to BV).

(18) History of severe allergic reactions or side-effects to granulocyte-colony stimulating factor (G-CSF) preparations, apheresis, or contrast agent.

(19) History of allergic reactions or side-effects to mouse-derived protein.

(20) History of allergic reactions or side-effects to iron or iron dextran.

(21) Pregnant or nursing, possibility of pregnancy, or planning on becoming pregnant before the end of the study.

(22) Participation in another clinical trial or clinical research within 6 months prior to providing consent.

(23) Patients whom the principal investigator or sub-investigator determines to be inappropriate on medical grounds.

**Supplementary Table S2**. The time course changes in the improvement rate from baseline to 52 weeks post-enrollment based on Child–Pugh score (FAS).

| Weeks  post-enrollment | CD34^+^ cell group | | | | SOC group | | | | Intergroup difference | | P-value |
| --- | --- | --- | --- | --- | --- | --- | --- | --- | --- | --- | --- |
|  |  |  |  |  |  |  |  |  | (CD34^+^ cell – SOC group) | |  |
|  | No. of patients | Improvement patients ^a)^ | Improvement rate (%) ^b)^ | | No. of patients | Improvement patients | Improvement rate (%) | | Point estimate | 95% CI |  |
|  |  |  | Point estimate | 95% CI |  |  | Point estimate | 95% CI |  |  |  |
| Baseline | 10 | － | － | － | 4 | － | － | － | － | － | － |
| Week 4 | 10 | 5 | 50.0 | (18.7, 81.3) | 4 | 1 | 25.0 | (0.6, 80.6) | 25.0 | (-37.9, 68.5) | 0.580 |
| Week 8 | 10 | 5 | 50.0 | (18.7, 81.3) | 4 | 1 | 25.0 | (0.6, 80.6) | 25.0 | (-37.9, 68.5) | 0.580 |
| Week 12 | 10 | 5 | 50.0 | (18.7, 81.3) | 4 | 0 | 0.0 | (0.0, 60.2) | 50.0 | (-16.8, 81.3) | 0.221 |
| Week 24 | 10 | 5 | 50.0 | (18.7, 81.3) | 4 | 1 | 25.0 | (0.6, 80.6) | 25.0 | (-37.9, 68.5) | 0.580 |
| Week 36 | 10 | 3 | 30.0 | (6.7, 65.2) | 3 | 1 | 25.0 | (0.6, 80.6) | 5.0 | (-55.7, 50.8) | 1.000 |
| Week 52 | 10 | 3 | 30.0 | (6.7, 65.2) | 3 | 0 | 0.0 | (0.0, 60.2) | 30.0 | (-29.9, 65.2) | 0.505 |
| a) Improvement was defined as a decrease in at least one point from baseline in the Child–Pugh score at each point.  b) Patients with missing measurements were defined as non-improving patients.  CI, confidence interval, SOC, standard-of-care; SVR, sustained virological response; FAS, full analysis set | | | | | | | | | | | |

**Supplementary Table S3**. The time course changes in the improvement rate from baseline to 52 weeks post-enrollment based on Child–Pugh class (FAS).

| Weeks  post-enrollment | CD34^+^ cell group | | | | SOC group | | | | Intergroup difference | | P-value |
| --- | --- | --- | --- | --- | --- | --- | --- | --- | --- | --- | --- |
|  |  |  |  |  |  |  |  |  | (CD34^+^ cell – SOC group) | |  |
|  | No. of patients | Improvement patients ^a)^ | Improvement  rate (%) ^b)^ | | No. of patients | Improvement patients | Improvement rate (%) | | Point estimate | 95% CI |  |
|  |  |  | Point estimate | 95% CI |  |  | Point estimate | 95% CI |  |  |  |
| Baseline | 10 | － | － | － | 4 | － | － | － | － | － | － |
| Week 4 | 10 | 4 | 40.0 | (12.2, 73.8) | 4 | 0 | 0.0 | (0.0, 60.2) | 40.0 | (-20.2, 73.8) | 0.251 |
| Week 8 | 10 | 3 | 30.0 | (6.7, 65.2) | 4 | 1 | 25.0 | (0.6, 80.6) | 5.0 | (-55.7, 50.8) | 1.000 |
| Week 12 | 10 | 4 | 40.0 | (12.2, 73.8) | 4 | 0 | 0.0 | (0.0, 60.2) | 40.0 | (-20.2, 73.8) | 0.251 |
| Week 24 | 10 | 5 | 50.0 | (18.7, 81.3) | 4 | 0 | 0.0 | (0.0, 60.2) | 50.0 | (-16.8, 81.3) | 0.221 |
| Week 36 | 10 | 2 | 20.0 | (2.5, 55.6) | 3 | 1 | 33.3 | (0.8, 90.6) | -13.3 | (-72.8, 37.9) | 1.000 |
| Week 52 | 10 | 2 | 20.0 | (2.5, 55.6) | 3 | 0 | 0.0 | (0.0, 70.8) | 20.0 | (-50.4, 56.5) | 1.000 |
| a) Improvement patients were defined as follows: change in Child–Pugh score from C to B, or C to A, or B to A at each point from baseline.  b) Patients with missing measurements were defined as non-improving patients.  CI, confidence interval, SOC, standard-of-care; SVR, sustained virological response; FAS, full analysis set | | | | | | | | | | | |

**Supplementary Table S4**. The time-course changes in the improvement rate from decompensated to compensated cirrhosis based on Child–Pugh score/class from baseline to 52 weeks post-enrollment (FAS).

| Weeks  post-enrollment | CD34^+^ cell group | | | | SOC group | | | | Intergroup difference | | P-value |
| --- | --- | --- | --- | --- | --- | --- | --- | --- | --- | --- | --- |
|  |  |  |  |  |  |  |  |  | (CD34^+^ cell – SOC group) | |  |
|  | No. of patients | Improvement patients ^a)^ | Improvement  rate (%) ^b)^ | | No. of patients | Improvement patients | Improvement rate (%) | | Point estimate | 95% CI |  |
|  |  |  | Point estimate | 95% CI |  |  | Point estimate | 95% CI |  |  |  |
| Baseline | 10 | － | － | － | 4 | － | － | － | － | － | － |
| Week 4 | 10 | 3 | 30.0 | (6.7, 65.2) | 4 | 0 | 0.0 | (0.0, 60.2) | 30.0 | (-29.9, 65.2) | 0.505 |
| Week 8 | 10 | 2 | 20.0 | (2.5, 55.6) | 4 | 1 | 25.0 | (0.6, 80.6) | -5.0 | (-61.4, 40.8) | 1.000 |
| Week 12 | 10 | 3 | 30.0 | (6.7, 65.2) | 4 | 0 | 0.0 | (0.0, 60.2) | 30.0 | (-29.9, 65.2) | 0.505 |
| Week 24 | 10 | 4 | 40.0 | (12.2, 73.8) | 4 | 0 | 0.0 | (0.0, 60.2) | 40.0 | (-20.2, 73.8) | 0.251 |
| Week 36 | 10 | 2 | 20.0 | (2.5, 55.6) | 3 | 1 | 33.3 | (0.8, 90.6) | -13.3 | (-72.8, 37.9) | 1.000 |
| Week 52 | 10 | 2 | 20.0 | (2.5, 55.6) | 3 | 0 | 0.0 | (0.0, 70.8) | 20.0 | (-50.4, 56.5) | 1.000 |
| a) Improvement patients were defined as follows: change in Child–Pugh score from C to A or B to A at each point from baseline.  b) Patients with missing measurements were defined as non-improving patients.  CI, confidence interval, SOC, standard-of-care; SVR, sustained virological response; FAS, full analysis set | | | | | | | | | | | |

**Supplementary Table S5**. Possible protocol treatment-related adverse events (AEs) during 52-week follow-up.

|  |  | |  | CD34^+^ cell group  No. patients (%) | | | | | |  | SOC group  No. patients (%) | | | | | |
| --- | --- | --- | --- | --- | --- | --- | --- | --- | --- | --- | --- | --- | --- | --- | --- | --- |
|  | Possible protocol treatment-related AEs | | | N=10 | | | | | |  | N=4 | | | | | |
|  |  |  |  | Mild | | Moderate | | Severe | |  | Mild | | Moderate | | Severe | |
|  |  | |  | n | (%) | n | (%) | n | (%) |  | n | (%) | n | (%) | n | (%) |
| **All adverse events** | | | | 9 | (90.0) | 0 | (0.0) | 0 | (0.0) |  | 0 | (0.0) | 0 | (0.0) | 0 | (0.0) |
| **G-CSF related** | | | | 7 | (70.0) | 0 | (0.0) | 0 | (0.0) |  | 0 | (0.0) | 0 | (0.0) | 0 | (0.0) |
|  | Blood and lymphatic system disorders | | | 1 | (10.0) | 0 | (0.0) | 0 | (0.0) |  | 0 | (0.0) | 0 | (0.0) | 0 | (0.0) |
|  |  | Splenomegaly | | 1 | (10.0) | 0 | (0.0) | 0 | (0.0) |  | 0 | (0.0) | 0 | (0.0) | 0 | (0.0) |
|  | General disorders and treatment site conditions | | | 3 | (30.0) | 0 | (0.0) | 0 | (0.0) |  | 0 | (0.0) | 0 | (0.0) | 0 | (0.0) |
|  |  | Fatigue | | 1 | (10.0) | 0 | (0.0) | 0 | (0.0) |  | 0 | (0.0) | 0 | (0.0) | 0 | (0.0) |
|  |  | Fever | | 2 | (20.0) | 0 | (0.0) | 0 | (0.0) |  | 0 | (0.0) | 0 | (0.0) | 0 | (0.0) |
|  | Clinical examination | | | 6 | (60.0) | 0 | (0.0) | 0 | (0.0) |  | 0 | (0.0) | 0 | (0.0) | 0 | (0.0) |
|  |  | Aspartate aminotransferase increased | | 1 | (10.0) | 0 | (0.0) | 0 | (0.0) |  | 0 | (0.0) | 0 | (0.0) | 0 | (0.0) |
|  |  | Lactate dehydrogenase increased | | 5 | (50.0) | 0 | (0.0) | 0 | (0.0) |  | 0 | (0.0) | 0 | (0.0) | 0 | (0.0) |
|  |  | Platelet count decreased | | 1 | (10.0) | 0 | (0.0) | 0 | (0.0) |  | 0 | (0.0) | 0 | (0.0) | 0 | (0.0) |
|  |  | Alkaline phosphatase increased | | 6 | (60.0) | 0 | (0.0) | 0 | (0.0) |  | 0 | (0.0) | 0 | (0.0) | 0 | (0.0) |
|  | Musculoskeletal and connective tissue disorders | | | 2 | (20.0) | 0 | (0.0) | 0 | (0.0) |  | 0 | (0.0) | 0 | (0.0) | 0 | (0.0) |
|  |  | Back pain | | 1 | (10.0) | 0 | (0.0) | 0 | (0.0) |  | 0 | (0.0) | 0 | (0.0) | 0 | (0.0) |
|  |  | Bone pain | | 1 | (10.0) | 0 | (0.0) | 0 | (0.0) |  | 0 | (0.0) | 0 | (0.0) | 0 | (0.0) |
| **Leukapheresis-related** | | | | 9 | (90.0) | 0 | (0.0) | 0 | (0.0) |  | 0 | (0.0) | 0 | (0.0) | 0 | (0.0) |
|  | Gastrointestinal disorders | | | 1 | (10.0) | 0 | (0.0) | 0 | (0.0) |  | 0 | (0.0) | 0 | (0.0) | 0 | (0.0) |
|  |  | Vomiting | | 1 | (10.0) | 0 | (0.0) | 0 | (0.0) |  | 0 | (0.0) | 0 | (0.0) | 0 | (0.0) |
|  | General disorders and treatment site conditions | | | 1 | (10.0) | 0 | (0.0) | 0 | (0.0) |  | 0 | (0.0) | 0 | (0.0) | 0 | (0.0) |
|  |  | Peripheral edema | | 1 | (10.0) | 0 | (0.0) | 0 | (0.0) |  | 0 | (0.0) | 0 | (0.0) | 0 | (0.0) |
|  | Hepatobiliary system disorders | | | 3 | (30.0) | 0 | (0.0) | 0 | (0.0) |  | 0 | (0.0) | 0 | (0.0) | 0 | (0.0) |
|  |  | Ascites | | 3 | (30.0) | 0 | (0.0) | 0 | (0.0) |  | 0 | (0.0) | 0 | (0.0) | 0 | (0.0) |
|  | Injury, poisoning, and treatment complications | | | 5 | (50.0) | 0 | (0.0) | 0 | (0.0) |  | 0 | (0.0) | 0 | (0.0) | 0 | (0.0) |
|  |  | Citric acid toxicity | | 5 | (50.0) | 0 | (0.0) | 0 | (0.0) |  | 0 | (0.0) | 0 | (0.0) | 0 | (0.0) |
|  | Clinical examination | | | 8 | (80.0) | 0 | (0.0) | 0 | (0.0) |  | 0 | (0.0) | 0 | (0.0) | 0 | (0.0) |
|  |  | Aspartate aminotransferase increased | | 1 | (10.0) | 0 | (0.0) | 0 | (0.0) |  | 0 | (0.0) | 0 | (0.0) | 0 | (0.0) |
|  |  | Albumin decreased | | 7 | (70.0) | 0 | (0.0) | 0 | (0.0) |  | 0 | (0.0) | 0 | (0.0) | 0 | (0.0) |
|  |  | Calcium decreased | | 1 | (10.0) | 0 | (0.0) | 0 | (0.0) |  | 0 | (0.0) | 0 | (0.0) | 0 | (0.0) |
|  |  | Cholesterol decreased | | 8 | (80.0) | 0 | (0.0) | 0 | (0.0) |  | 0 | (0.0) | 0 | (0.0) | 0 | (0.0) |
|  |  | Cholinesterase decreased | | 7 | (70.0) | 0 | (0.0) | 0 | (0.0) |  | 0 | (0.0) | 0 | (0.0) | 0 | (0.0) |
|  |  | Lactate Dehydrogenase increased | | 2 | (20.0) | 0 | (0.0) | 0 | (0.0) |  | 0 | (0.0) | 0 | (0.0) | 0 | (0.0) |
|  |  | Potassium decreased | | 2 | (20.0) | 0 | (0.0) | 0 | (0.0) |  | 0 | (0.0) | 0 | (0.0) | 0 | (0.0) |
|  |  | Blood pressure decreased | | 2 | (20.0) | 0 | (0.0) | 0 | (0.0) |  | 0 | (0.0) | 0 | (0.0) | 0 | (0.0) |
|  |  | High-density lipoprotein decreased | | 2 | (20.0) | 0 | (0.0) | 0 | (0.0) |  | 0 | (0.0) | 0 | (0.0) | 0 | (0.0) |
|  |  | Low-density lipoprotein decreased | | 4 | (40.0) | 0 | (0.0) | 0 | (0.0) |  | 0 | (0.0) | 0 | (0.0) | 0 | (0.0) |
|  |  | Platelet count decreased | | 3 | (30.0) | 0 | (0.0) | 0 | (0.0) |  | 0 | (0.0) | 0 | (0.0) | 0 | (0.0) |
|  |  | Total protein decreased | | 3 | (30.0) | 0 | (0.0) | 0 | (0.0) |  | 0 | (0.0) | 0 | (0.0) | 0 | (0.0) |
|  |  | Body weight increased | | 1 | (10.0) | 0 | (0.0) | 0 | (0.0) |  | 0 | (0.0) | 0 | (0.0) | 0 | (0.0) |
|  |  | Alkaline phosphatase increased | | 1 | (10.0) | 0 | (0.0) | 0 | (0.0) |  | 0 | (0.0) | 0 | (0.0) | 0 | (0.0) |
|  |  | Creatinine phosphokinase decreased | | 2 | (20.0) | 0 | (0.0) | 0 | (0.0) |  | 0 | (0.0) | 0 | (0.0) | 0 | (0.0) |
| **Cell transplant procedure-related** | | | | 1 | (10.0) | 0 | (0.0) | 0 | (0.0) |  | 0 | (0.0) | 0 | (0.0) | 0 | (0.0) |
|  | General disorders and treatment site conditions | | | 1 | (10.0) | 0 | (0.0) | 0 | (0.0) |  | 0 | (0.0) | 0 | (0.0) | 0 | (0.0) |
|  |  | fever | | 1 | (10.0) | 0 | (0.0) | 0 | (0.0) |  | 0 | (0.0) | 0 | (0.0) | 0 | (0.0) |
| **Others** | | | | 2 | (20.0) | 0 | (0.0) | 0 | (0.0) |  | 0 | (0.0) | 0 | (0.0) | 0 | (0.0) |
|  | Clinical examination | | | 1 | (10.0) | 0 | (0.0) | 0 | (0.0) |  | 0 | (0.0) | 0 | (0.0) | 0 | (0.0) |
|  |  | Alkaline phosphatase increased | | 1 | (10.0) | 0 | (0.0) | 0 | (0.0) |  | 0 | (0.0) | 0 | (0.0) | 0 | (0.0) |
|  | Renal and urinary tract disorders | | | 1 | (10.0) | 0 | (0.0) | 0 | (0.0) |  | 0 | (0.0) | 0 | (0.0) | 0 | (0.0) |
|  |  | Hematuria | | 1 | (10.0) | 0 | (0.0) | 0 | (0.0) |  | 0 | (0.0) | 0 | (0.0) | 0 | (0.0) |

SOC, standard-of-care; G-CSF, granulocyte-colony stimulating factor

**Supplementary Table S6**. The time-course changes from baseline to 52 weeks post-enrollment in albumin-bilirubin (ALBI) score (patients with SVR).

| Weeks  post-enrollment | CD34^+^ cell group | | | | SOC group | | | | Intergroup difference | | P-value |
| --- | --- | --- | --- | --- | --- | --- | --- | --- | --- | --- | --- |
|  |  |  |  |  |  |  |  |  | (CD34^+^ cell –  SOC group) | |  |
|  | No. of patients | LSMean ^a)^ | | | No. of patients | LSMean | | | Point estimate | 95% CI |  |
|  |  | Point estimate | SE | 95% CI |  | Point estimate | SE | 95% CI |  |  |  |
| Baseline | 8 | － | － | － | 2 | － | － | － | － | － | － |
| Week 4 | 8 | 0.08 | 0.07 | (-0.08, 0.23) | 2 | -0.07 | 0.13 | (-0.38, 0.24) | 0.15 | ( -0.20, 0.50) | 0.350 |
| Week 8 | 8 | -0.03 | 0.06 | (-0.17, 0.11) | 2 | -0.14 | 0.12 | (-0.43, 0.14) | 0.11 | ( -0.20, 0.43) | 0.437 |
| Week 12 | 8 | -0.13 | 0.07 | (-0.29, 0.03) | 2 | 0.00 | 0.13 | (-0.31, 0.32) | -0.13 | ( -0.48, 0.22) | 0.405 |
| Week 24 | 8 | -0.13 | 0.07 | (-0.29, 0.03) | 2 | 0.10 | 0.14 | (-0.22, 0.42) | -0.23 | ( -0.58, 0.13) | 0.179 |
| Week 36 | 8 | -0.06 | 0.07 | (-0.22, 0.09) | 2 | 0.04 | 0.14 | (-0.28, 0.35) | -0.10 | ( -0.45, 0.25) | 0.534 |
| Week 52 | 8 | -0.07 | 0.08 | (-0.26, 0.13) | 2 | -0.11 | 0.17 | (-0.50, 0.28) | 0.05 | ( -0.39, 0.48) | 0.814 |

1. Least Squares Means (LSMean) by MMRM.

SE, standard error; CI, confidence interval; SOC, standard-of-care; SVR, sustained virological response; MMRM, mixed models for repeated measures.

**Supplementary Table S7**. The time-course changes in the improvement rate from baseline to 52 weeks post-enrollment based on Child–Pugh score (patients with SVR).

| Weeks  post-enrollment | CD34^+^ cell group | | | | SOC group | | | | Intergroup difference | | P-value |
| --- | --- | --- | --- | --- | --- | --- | --- | --- | --- | --- | --- |
|  |  |  |  |  |  |  |  |  | (CD34^+^ cell –SOC group) | |  |
|  | No. of patients | Improvement patients ^a)^ | Improvement  rate (%) ^b)^ | | No. of patients | Improvement patients | Improvement  rate (%) | | Point estimate | 95% CI |  |
|  |  |  | Point estimate | 95% CI |  |  | Point estimate | 95% CI |  |  |  |
| Baseline | 8 | － | － | － | 2 | － | － | － | － | － | － |
| Week 4 | 8 | 5 | 62.5 | (24.5, 91.5) | 2 | 1 | 50.0 | (1.3, 98.7) | 12.5 | (-55.2, 75.5) | 1.000 |
| Week 8 | 8 | 4 | 50.0 | (15.7, 84.3) | 2 | 1 | 50.0 | (1.3, 98.7) | 0.0 | (-65.8, 65.8) | 1.000 |
| Week 12 | 8 | 5 | 62.5 | (24.5, 91.5) | 2 | 0 | 0.0 | (0.0, 84.2) | 62.5 | (-24.7, 92.2) | 0.444 |
| Week 24 | 8 | 5 | 62.5 | (24.5, 91.5) | 2 | 1 | 50.0 | (1.3, 98.7) | 12.5 | (-55.2, 75.5) | 1.000 |
| Week 36 | 8 | 3 | 37.5 | (8.5, 75.5) | 2 | 1 | 50.0 | (1.3, 98.7) | -12.5 | (-75.5, 55.2) | 1.000 |
| Week 52 | 8 | 3 | 37.5 | (8.5, 75.5) | 2 | 0 | 0.0 | (0.0, 84.2) | 37.5 | (-48.4, 76.5) | 1.000 |
| a) Improvement was defined as a decrease in at least one point from baseline in the Child–Pugh score at each point.  b) Patients with missing measurements were defined as non-improving patients.  CI, confidence interval, SOC, standard-of-care; SVR, sustained virological response | | | | | | | | | | | |

**Supplementary Table S8**. The time-course changes in the improvement rate from baseline to 52 weeks post-enrollment based on Child–Pugh class (patients with SVR).

| Weeks  post-enrollment | CD34^+^ cell group | | | | SOC group | | | | Intergroup difference | | P-value |
| --- | --- | --- | --- | --- | --- | --- | --- | --- | --- | --- | --- |
|  |  |  |  |  |  |  |  |  | (CD34^+^ cell – SOC group) | |  |
|  | No. of patients | Improvement patients ^a)^ | Improvement  rate (%) ^b)^ | | No. of patients | Improvement patients | Improvement  rate (%) | | Point estimate | 95% CI |  |
|  |  |  | Point estimate | 95% CI |  |  | Point estimate | 95% CI |  |  |  |
| Baseline | 8 | － | － | － | 2 | － | － | － | － | － | － |
| Week 4 | 8 | 4 | 50.0 | (15.7, 84.3) | 2 | 0 | 0.0 | (0.0, 84.2) | 50.0 | (-36.9, 85.0) | 0.467 |
| Week 8 | 8 | 3 | 37.5 | (8.5, 75.5) | 2 | 1 | 50.0 | (1.3, 98.7) | -12.5 | (-75.5, 55.2) | 1.000 |
| Week 12 | 8 | 4 | 50.0 | (15.7, 84.3) | 2 | 0 | 0.0 | (0.0, 84.2) | 50.0 | (-36.9, 85.0) | 0.467 |
| Week 24 | 8 | 5 | 62.5 | (24.5, 91.5) | 2 | 0 | 0.0 | (0.0, 84.2) | 62.5 | (-24.7, 92.2) | 0.444 |
| Week 36 | 8 | 2 | 25.0 | (3.2, 65.1) | 2 | 1 | 50.0 | (1.3, 98.7) | -25.0 | (-84.2, 43.6) | 1.000 |
| Week 52 | 8 | 2 | 25.0 | (3.2, 65.1) | 2 | 0 | 0.0 | (0.0, 84.2) | 25.0 | (-59.2, 66.8) | 1.000 |
| a) Improvement patients were defined as follows: change in Child–Pugh score from C to B, C to A, or B to A at each point from baseline.  b) Patients with missing measurements were defined as non-improving patients.  CI, confidence interval, SOC, standard-of-care; SVR, sustained virological response | | | | | | | | | | | |

**Supplementary Table S9**. The time-course changes in the improvement rate from decompensated to compensated cirrhosis based on Child–Pugh score/class from baseline to 52 weeks post-enrollment (patients with SVR).

| Weeks  post-enrollment | CD34^+^ cell group | | | | SOC group | | | | Intergroup difference | | P-value |
| --- | --- | --- | --- | --- | --- | --- | --- | --- | --- | --- | --- |
|  |  |  |  |  |  |  |  |  | (CD34^+^ cell – SOC group) | |  |
|  | No. of patients | Improvement patients ^a)^ | Improvement  rate (%) ^b)^ | | No. of patients | Improvement patients | Improvement  rate (%) | | Point estimate | 95% CI |  |
|  |  |  | Point estimate | 95% CI |  |  | Point estimate | 95% CI |  |  |  |
| Baseline | 8 | － | － | － | 2 | － | － | － | － | － | － |
| Week 4 | 8 | 3 | 37.5 | (8.5, 75.5) | 2 | 0 | 0.0 | (0.0, 84.2) | 37.5 | (-48.4, 76.5) | 1.000 |
| Week 8 | 8 | 2 | 25.0 | (3.2, 65.1) | 2 | 1 | 50.0 | (1.3, 98.7) | -25.0 | (-84.2, 43.6) | 1.000 |
| Week 12 | 8 | 3 | 37.5 | (8.5, 75.5) | 2 | 0 | 0.0 | (0.0, 84.2) | 37.5 | (-48.4, 76.5) | 1.000 |
| Week 24 | 8 | 4 | 50.0 | (15.7, 84.3) | 2 | 0 | 0.0 | (0.0, 84.2) | 50.0 | (-36.9, 85.0) | 0.467 |
| Week 36 | 8 | 2 | 25.0 | (3.2, 65.1) | 2 | 1 | 50.0 | (1.3, 98.7) | -25.0 | (-84.2, 43.6) | 1.000 |
| Week 52 | 8 | 2 | 25.0 | (3.2, 65.1) | 2 | 0 | 0.0 | (0.0, 84.2) | 25.0 | (-59.2, 66.8) | 1.000 |
| a) Improvement patients were defined as follows: change in Child–Pugh score from C to A or B to A at each point from baseline.  b) Patients with missing measurements were defined as non-improving patients.  CI, confidence interval, SOC, standard-of-care; SVR, sustained virological response | | | | | | | | | | | |
